# Intra-QT Spectral Coherence as a Possible Noninvasive Marker of Sustained Ventricular Tachycardia

**DOI:** 10.1155/2014/583035

**Published:** 2014-07-15

**Authors:** Gianfranco Piccirillo, Federica Moscucci, Alessandro Persi, Daniele Di Barba, Maria Antonella Pappadà, Pietro Rossi, Raffaele Quaglione, Bich Lien Nguyen, Francesco Barillà, Matteo Casenghi, Damiano Magrì

**Affiliations:** ^1^Dipartimento di Scienze Cardiovascolari, Respiratorie, Nefrologiche, Anestesiologiche e Geriatriche, Policlinico Umberto I, “Sapienza” University of Rome, Viale del Policlinico No. 155, 00185 Roma, Italy; ^2^Division of Cardiology, S. Giovanni Calibita Fatebenefratelli Hospital, Isola Tiberina, Piazza Ponte dei Quattro Capi, 39 186 Roma, Italy; ^3^Dipartimento di Medicina Clinica e Molecolare, S. Andrea Hospital, “Sapienza” University of Rome, Via di Grottarossa 1035/1039, 00189 Roma, Italy

## Abstract

Sudden cardiac death is the main cause of mortality in patients affected by chronic heart failure (CHF) and with history of myocardial infarction. No study yet investigated the intra-QT phase spectral coherence as a possible tool in stratifying the arrhythmic susceptibility in patients at risk of sudden cardiac death (SCD). We, therefore, assessed possible difference in spectral coherence between the ECG segment extending from the *q* wave to the* T* wave peak (QT_*p*_) and the one from* T* wave peak to the* T* wave end (*T*_*e*_) between patients with and without Holter ECG-documented sustained ventricular tachycardia (VT). None of the QT variability indexes as well as most of the coherences and RR power spectral variables significantly differed between the two groups except for the QT_*p*_-*T*_*e*_ spectral coherence. The latter was significantly lower in patients with sustained VT than in those without (0.508 ± 0.150 versus 0.607 ± 0.150, *P* < 0.05). Although the responsible mechanism remains conjectural, the QT_*p*_-*T*_*e*_ spectral coherence holds promise as a noninvasive marker predicting malignant ventricular arrhythmias.

## 1. Introduction

A prospective multicenter study indicates the myocardial temporal repolarization dispersion, measured with the beat-to-beat QT variability index (QTVI), as a marker predicting cardiac death without specifically predicting sudden cardiac death (SCD) in patients with chronic heart failure (CHF) [1]. A possible explanation might be that QTVI results in a better electrocardiographic (ECG) marker for the severity of CHF than for SCD, being closely influenced also by the severity of left ventricular dysfunction and neurohumoral activation [2–5]. Nonetheless, the QTVI is customarily calculated together with another marker that assesses changes in myocardial temporal repolarization, namely, the spectral coherence between the QT and RR intervals [2–5]. The QT-RR spectral coherence uses a value ranging from 0 to 1 to express whether the two ECG signals yield coherent oscillations over time, and it reaches maximum when QT and RR interval oscillations proportionally correspond [2–5]. Unfortunately, this specific index is more complex to calculatethan the simple correlation between the myocardial action potential duration and the diastolic interval expressed with ventricular electrical restitution curves. Indeed, QT-RR interval spectral coherence evaluates temporal patterns in oscillating signals whereas regression curves or lines measure QT variations as a function of RR interval variations. In healthy subjects, QT-RR interval spectral coherence tends to remain relatively low (less than 0.400) but it logically tends to decrease further in CHF (less than 0.300) [2–5]. Because this index, as well as the QTVI, reflects many confounding factors, scarce data are available on its ability to predict cardiovascular mortality. To our knowledge, only one study has observed that QT-RR interval spectral coherence might be able to predict SCD in females with CHF [6]. More recently, our research group focused attention on temporal myocardial dispersion in the two segments comprised in the whole QT interval, namely, the segment extending from the* q* wave to the* T* wave peak (QT_*p*_) and the one from the* T* wave peak to the* T* wave end (*T*_*e*_) [4, 5, 7]. We also analyzed the QT_*p*_-RR and the* T*_*e*_-RR spectral coherence but both failed to be associated with the risk of SCD [4, 5]. However, no information yet shows whether the intra-QT spectral coherence, namely, that between QT_*p*_ and* T*_*e*_, could be associated with sustained ventricular tachycardia (VT) and, possibly, with an increased SCD risk.

In this study, extending our previous research on QT-RR spectral coherence as a possible noninvasive marker for SCD, we sought to investigate possible relationship between QT_*p*_-*T*_*e*_ spectral coherence and ventricular arrhythmias propensity in a study sample yet considered at moderate-to-high risk for SCD. To do so, we calculated the spectral coherence between QT_*p*_ and* T*_*e*_ in short-term ECG recordings from patients with and without Holter ECG-documented VT.

## 2. Methods

### 2.1. Study Subjects

We analyzed short-term (5-minute) ECG recordings from clinically stable outpatients referred to our cardiology unit with a history of myocardial infarction or with systolic dysfunction related to primary or ischemic dilated cardiomyopathy. We defined clinically stable patients as those who had not been hospitalized or had their therapy adjusted or had experienced any other acute coronary artery or noncoronary event during the past three months. All participants with known coronary disease had undergone revascularization either cutaneously or by aortocoronary artery bypass at least 3 months before the study. None of the patients had malignancy, primary valve disease, atrial fibrillation, numerous premature beats (one premature beat per minute was permitted), or other arrhythmias likely to interfere with RR and QT analysis. None of the patients was in New York Heart Association (NYHA) class IV. Patients were retrospectively grouped according to the presence or absence of sustained VT, defined as a ventricular rhythm lasting more than 30 seconds, on a 24-hour Holter ECG recording executed within a month from the short-term ECG recording.

A total of 124 short-term (5-minute) ECG recordings were selected for the present study purpose, 52 ECG recordings from postmyocardial infarction patients (mean left ventricular ejection fraction of 47 ± 7%) and 72 ECG recordings owing to patients with dilated cardiomyopathy (54 with postischemic and 18 with primitive etiology, mean left ventricular ejection fraction of 32 ± 6%). Within the analyzed dataset, 27 ECG recordings were from patients with Holter ECG-documented VT (9 from patients with previous myocardial infarction and 14 from patients with postischemic and 4 from patients with primitive dilated cardiomyopathy) and 97 from those without. Thus, patients with a history of ischemic cardiomyopathy were 106 and in this group 23 developed VT. In the group of primitive cardiomyopathy, VT arose in 4 patients with the same incidence of the other group (22%) (Table 1).

The study complied with the ethical rules for human experimentation stated in the Declaration of Helsinki.

### 2.2. Study Protocol and Offline Data Analysis

After a 15-minute rest lying down, each subject underwent a 5-minute, single ECG lead recordingduring controlled breathing (15 breaths per minute, 0.25 Hz). ECG signals were acquired and digitalized with custom-designed card (National Instruments USB-6008, Austin, Texas, USA) at a sampling frequency of 500 Hz. Software for data acquisition, storage, and analysis was designed and produced by our research group with LabView program (National Instruments USB-6008, Austin, Texas, USA) and it is described in detail elsewhere [3, 5, 8–10]. All digitized signal recordings were checked by a single physician [G.P.] blinded to subjects circumstances.

**Figure 1:**
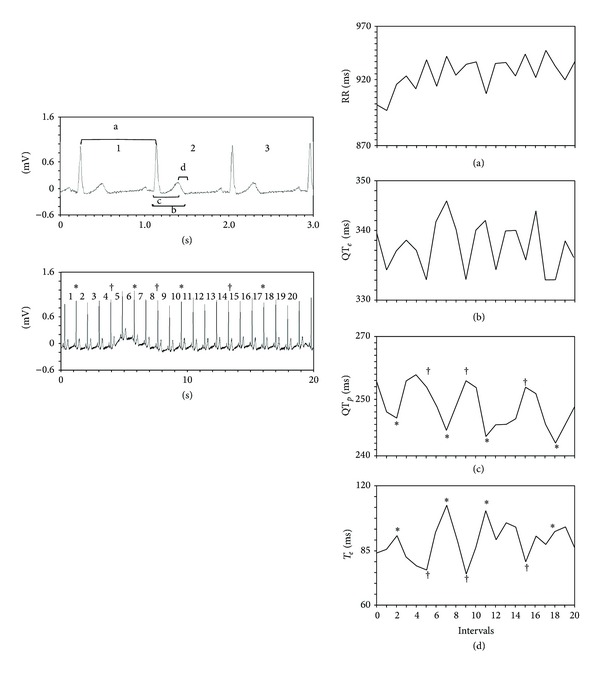
The upper left panel shows the four intervals studied: a = RR; b = QT_*e*_; c = QT_*p*_; d =* T*_*e*_. The lower left panel shows a beat-to-beat ECG recording extended to include the first 20 beats with the relative RR intervals. The right panels give the RR (a), QT_*e*_ (b), QT_*p*_ (c), and* T*_*e*_ (d) intervals measured on the 20 beats shown in the lower left panel. The asterisks (∗) in this panel indicate the second, seventh, eleventh, and eighteenth beats, shown also in panels (c) and (d). In panels (c) and (d) the four lowest QT_*p*_ with an asterisk correspond to four interval peaks* T*_*e*_. The daggers (†) show the fifth, ninth, and fifteenth beats that vary in line with the three highest QT_*p*_ values and correspond to the three lowest* T*_*e*_ values. This pattern indicates that the two ECG signals oscillate in a substantially coherent manner. Conversely, the RR intervals yield poor coherence (a) with the other intervals studied.

**Figure 2:**
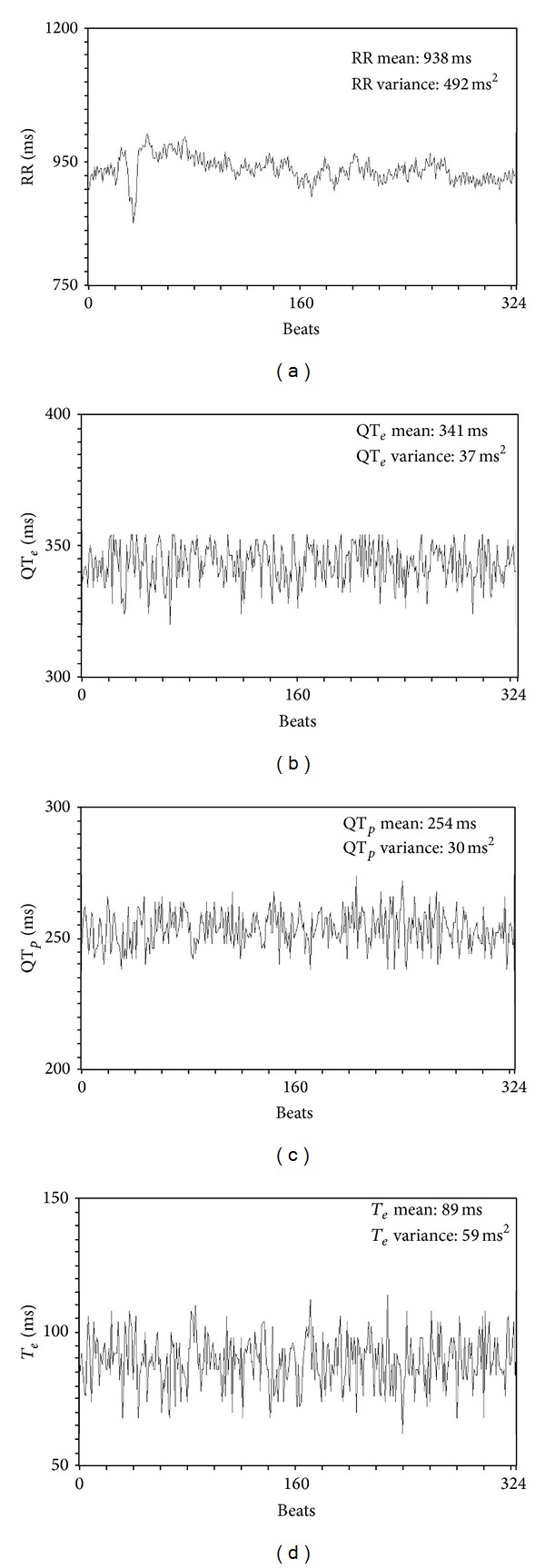
Median and variance in a short-term (5-minute) ECG recording of the studied variables, RR (a), QT_*e*_ (b), QT_*p*_ (c), and* T*_*e*_ (d).

The following intervals from the respective beat-to-beat ECG recordings have been measured: RR, QT_*e*_ (from the *q* wave to the *T* wave end), QT_*p*_ (from the *q* wave to the *T* wave peak), and* T*_*e*_ (difference between QT_*e*_ and QT_*p*_) (Figure 1). We therefore calculated mean and variance values for each of these intervals and then we used the original formula proposed by Berger et al. [2] to calculate three different QT variability indexes [4, 5, 7] (Figure 2):

(1) $$\begin{array}{c} {\text{QT}}_{e}\mathrm{VI}=\log_{10}\left\{\frac{\left[\left[\text{Q}{\text{T}}_{e}\;\text{variance}\right]/{\left[{\text{QT}}_{e}\;\text{mean}\right]}^{2}\right]}{\left[\left[\text{RR}\;\text{variance}\right]/{\left[\text{RR}\;\text{mean}\right]}^{2}\right]}\right\}\\ {\text{QT}}_{p}\mathrm{VI}=\log_{10}\left\{\frac{\left[\left[{\text{QT}}_{p}\;\text{variance}\right]/{\left[{\text{QT}}_{p}\;\text{mean}\right]}^{2}\right]}{\left[\left[\text{RR}\;\text{variance}\right]/{\left[RR\;\text{mean}\right]}^{2}\right]}\right\}\\ T_{e}\mathrm{VI}=\log_{10}\left\{\frac{\left[\left[T_{e}\;\text{variance}\right]/{\left[T_{e}\;\text{mean}\right]}^{2}\right]}{\left[\left[\text{RR}\;\text{variance}\right]/{\left[\text{RR}\;\text{mean}\right]}^{2}\right]}\right\}. \end{array}$$

**Figure 3:**
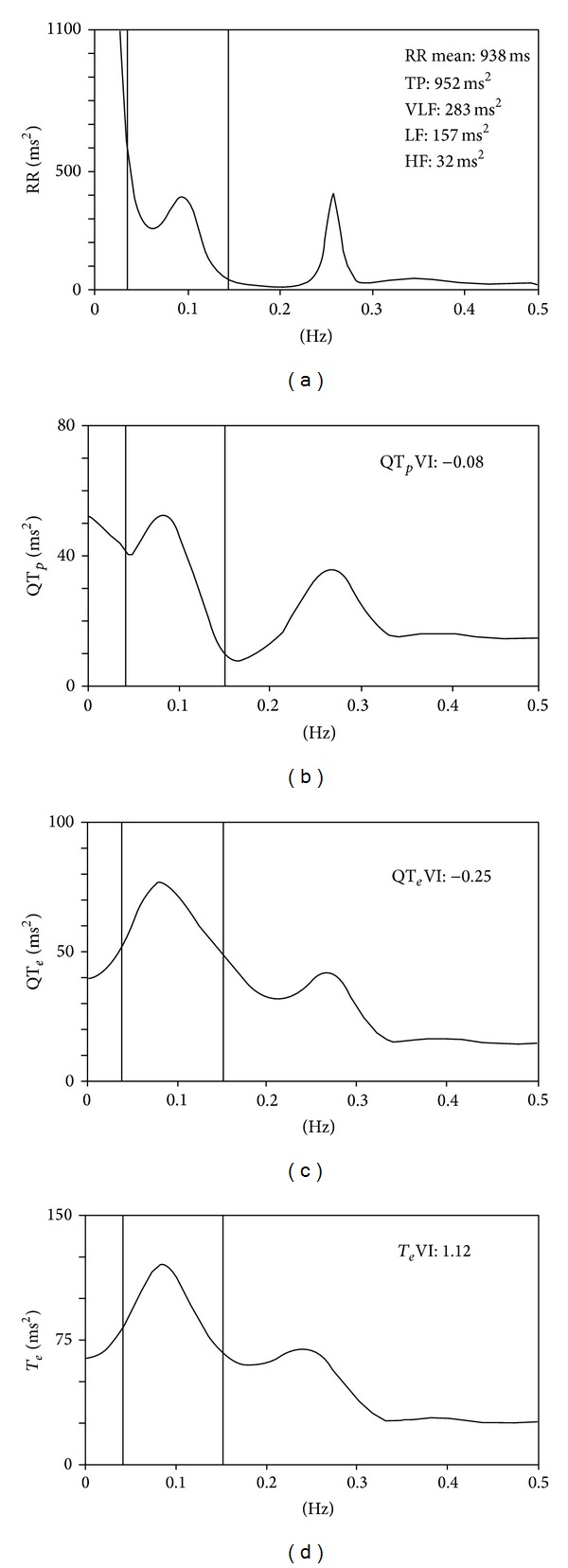
Power spectral analysis of RR (a), QT_*e*_ (b), QT_*p*_ (c), and* T*_*e*_ (d) calculated on a short-term (5-minute) ECG recording from the same patient shown in Figure 2. All the power spectra contain a high-frequency power component synchronous with breathing (between 0.20 and 0.30 Hz) and low-frequency power around 0.10 Hz.

From the same 5-minute ECG segments we also determined the total power (TP) of RR intervals [8, 11]. For RR intervals we calculated the following spectral components: high-frequency (HF) power (from 0.15 to 0.40 Hz), low-frequency (LF) power (from 0.04 to 0.15 Hz Eq), and very-low-frequency (VLF) power (below 0.04 Hz Eq) [8, 9] (Figure 3).

**Figure 4:**
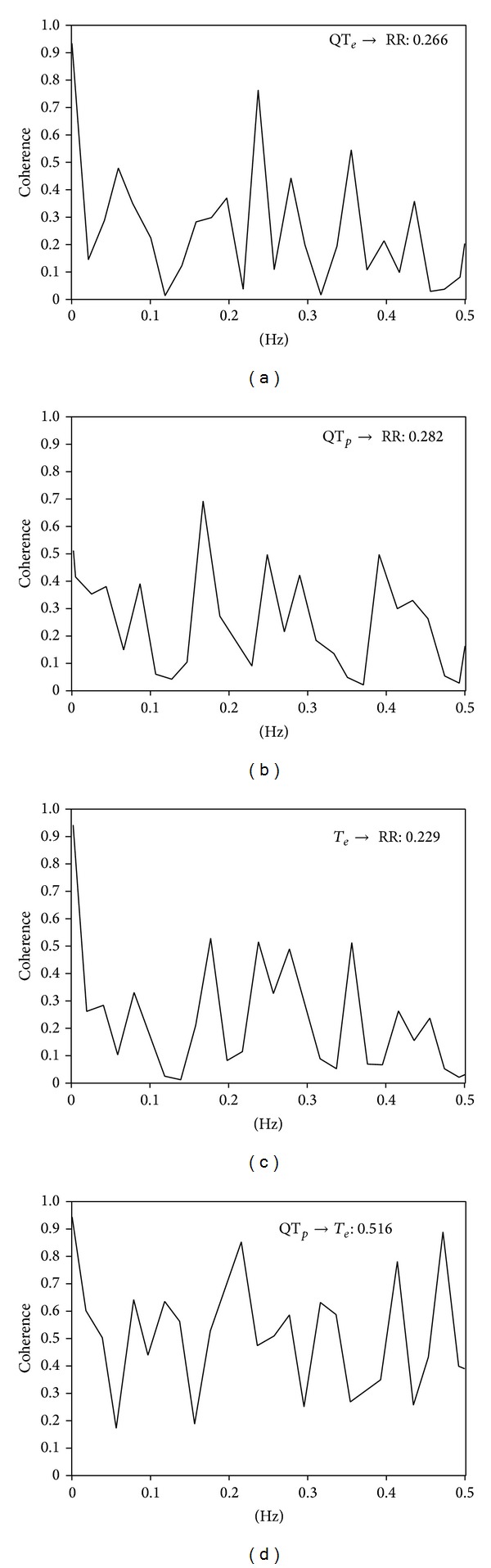
Spectral coherence between the tested variables in a patient with chronic heart failure: QT_*e*_→RR (a), QT_*p*_→RR (b),* T*_*e*_→RR (c), and *e*QT_*p*_→*T*_*e*_ (d). Spectral coherence reaches maximum in QT_*p*_→*T*_*e*_.

The same ECG intervals were also used for power spectral analysis with an autoregressive algorithm also for QT_*e*_, QT_*p*_, and* T*_*e*_ intervals (Figure 1). Cross spectral analysis was thenused to evaluate the reciprocal influence (coherence function) between RR, QT_*e*_, QT_*p*_, and* T*_*e*_ [3–5] (Figure 4). Coherence expresses the fraction of power at a given frequency in either time series and is explained as a linear transformation of the other thus providing an index of a linear association between the two signals. The coherence function *γ*(*f*) was then computed according to the following formula [2–5] (Figure 4):

(2) $$\begin{array}{c} \gamma \left[f\right]=\frac{{\left|P_{xy}\left[f\right]\right|}^{2}}{Pxx\left[f\right]Pyy\left[f\right]}, \end{array}$$

where *f* is frequency, *Pxx*[*f*] is the RR intervalspectrum, *Pyy*[*f*] are the QT_*e*_ or QT_*p*_ or* T*_*e*_ interval spectra, and *Pxy*[*f*] is the cross spectrum. The coherence function measuresthe degree of linear interaction between RR and QT_*e*_ or QT_*p*_ or* T*_*e*_ interval oscillations as a function of their frequency. The coherence function value ranges between zero and one. Mean coherences were measured by averaging *γ*[*f*] over the frequency bands from 0 to 0.50 Hz.

Last, from the same 5-minute ECG segment, the corrected QT_*e*_, QT_*p*_, and* T*_*e*_ intervals were obtained according to the formulas proposed by Bazett (QT_*e*_/RR^0.5^; QT_*p*_/RR^0.5^;* T*_*e*_/RR^0.5^), Friedericia (QT_*e*_/RR^0.33^; QT_*p*_/RR^0.33^;* T*_*e*_/RR^0.33^), Lilly (QT_*e*_/RR^0.4^; QT_*p*_/RR^0.4^;* T*_*e*_/RR^0.4^), and Framingham (QT_*e*_ + [0.154∗{1000-RR}]; QT_*p*_ + [0.154∗{1000-RR}];* T*_*e*_ + [0.154∗{1000-*RR*}]).

### 2.3. Statistical Analysis

Unless otherwise indicated all data are expressed as mean ± SD. Data with skeweddistribution are given as median and interquartile range [75th percentile–25th percentile]. Categorical variables were analysed with the *χ* ^2^ test. Unpaired *t*-test was used to compare data for the normally distributed variables. Mann-Whitney *U* test was used to compare nonnormally distributed variables (as previously evaluated by Kolmogorov-Smirnov test).

Stepwise multiple regression analysis was used to assess the relationship between the different coherence variables and the other spectral and nonspectral variables studied. Finally, multivariate logistic regression analysis (adjusted for age, sex, blood pressure, heart rate, left ventricular ejection fraction, and treatment) was used to determine whether the different variables were independently associated with the Holter ECG-documented sustained VT. *P* values of less than or equal to 0.05 were considered to indicate statistical significance. All data were evaluated with the database SPSS-PC+ [SPSS-PC+ Inc., Chicago, Illinois].

## 3. Results

Neither age, body mass index (BMI), gender distribution, heart rate, systemic arterial pressures, NYHA class, coronary disease prevalence, drug therapy, nor corrected values for QT_*e*_, QT_*p*_, or* T*_*e*_ differed between the two study groups (Table 1). No significant differences were found in any myocardial temporal dispersion indexes (Table 2) or most spectral coherences variables (QT_*e*_-RR, QT_*p*_-RR,* T*_*e*_-RR, QT_*e*_-QT_*p*_, and QT_*e*_-*T*_*e*_). Conversely, the QT_*p*_-*T*_*e*_ spectral coherence was significantly lower in patients with documented VTs than in those without (*P* < 0.05) (Table 3). No significant differences were found in RR power spectral analysis components (TP, VLF, LF, and HF) while LF/HF ratio was significantly lower in the VT group than in the counterpart (see Table 4).

Multivariate logistic regression identified as the only variable predicting VT QT_*p*_-*T*_*e*_ coherence. The unadjusted odds ratio was 0.05 (95% CI 0.004–0.61), *P* < 0.05, and adjusted odds ratio was 0.05 (95% CI 0.01–0.77), *P* < 0.05. We found no correlation between QT_*p*_-*T*_*e*_ spectral coherence and the other tested spectral and nonspectral variables.

## 4. Discussion

Our preliminary and retrospective study suggests that the intra-QT coherence analysis, as assessed by the QT_*p*_-*T*_*e*_ spectral coherence, might be a useful noninvasive predictor for VT in patients yet at moderate-to-high risk for SCD. Indeed, spectral coherence between QT_*p*_ and* T*_*e*_ was significantly lower in patients with Holter ECG-documented VT than in those without. Conversely, no other noninvasive myocardial temporal repolarization dispersion variable differed in these two groups. Studying patients with and without documentedsustained VT, regardless of their left ventricular ejection fraction, allowed us to compare spectral coherence in patients with a possible different propensity to malignant ventricular arrhythmias and, most likely, to SCD. Indeed, it is reported that those subjects with VT have an almost twofold higher risk of SCD than postmyocardial infarction patients with or without systolic dysfunction and, accordingly, are eligible for a cardioverter defibrillator implantation [12].

The possible mechanisms responsible for altering QT_*p*_-*T*_*e*_ spectral coherence remain conjectural. The two ECG intervals we analyzed, namely, the QT_*p*_ and* T*_*e*_, undoubtedly differ in their electrophysiological meaning. Despite remaining controversial [13–15], some investigators consider that the QT_*p*_ depends on the action potential duration only in the epicardial layer [16, 17]. Conversely, the* T*_*e*_ predominantly measures myocardial repolarization in the M-cell layer and also in the layers in which depolarization lasts longer. Thus, the* T*_*e*_ is thought to reflect the maximum difference in repolarization between the myocardial layers and it has been suggested as a pure noninvasive marker of transmural dispersion repolarization [15, 16]. Notably, the* T*_*e*_ interval is mainly influenced by the terminal part of the action potential, namely, from the rapidly (I_*Kr*_) and slowly (I_*Ks*_) activating components, as well as from the delayed rectifier current and the inward rectifier current (I_*K1*_), whereas the QT_*p*_ interval reasonably depends on the oscillation in the depolarization phase and thus on inward Na (*I*_Na_) currents, as well as on the early repolarization phase, mainly under the transient *K* outward (*I*_to_) control and the sarcoplasmatic reticulum Ca uptake (*I*_up_) currents [17–19]. Whatever malfunction of this complex ion channels network could delay or alter one of these two QT segments, reducing their spectral coherence and increasing the malignant ventricular arrhythmias susceptibility [18, 19]. Another possible explanation of our finding might come from the long-standing evidence that VT originates from reentrant circuits created by structural or functional anomalies in myocardial tissue. Indeed, myocardial zones able to form reentrant circuits might induce temporal dishomogeneity in various myocardial action potential components. Accordingly, these changes, generating a low ability for synchronous QT_*p*_ and* T*_*e*_ interval oscillations, might result in low spectral coherence between these two variables. Last, it should be underlined that the original work by Berger et al. in 1997 [2] contains two major statements on spectral coherence. The first is that identical oscillations in the RR interval spectrum, and therefore in the respiratory spectrum, appear also in the myocardial repolarization spectrum. This statement find confirmation in this study as well as in another one we made [20]. The second states that the spectrum over 0.20 Hz contains no major oscillations, thus justifying a calculation of spectral coherence up to this frequency. However, in two-thirds of patients with advance CHF, ECG recordings obtained in patients lying down show an abnormal, spontaneous slow respiratory rhythm (periodic or Cheyne-Stokes breathing), these oscillations preferentially altering the spectral VLF power component (below 0.04 Hz) of the RR interval [21, 22]. To make RR and QT interval variability more homogenous, we asked patients to breathe at a fixed respiratory rate, namely, 15 breaths per minute [23–25]. In this way respiration forms a homogeneous power component around 0.25 Hz (HF power) that leaves the other power spectral components unaltered (Figure 3). Accordingly, to evaluate spectral coherence influenced by breathing and to make sure that no oscillations were missed, wewidened the spectral window from 0 to 0.50 Hz.

Interestingly, a possible advantage of QT_*p*_-*T*_*e*_ spectral coherence is that this variable is completely independent of heart rate, corrected or uncorrected action potential duration, left ventricular ejection fraction, age, and all the other spectral and nonspectral variables studied. Conversely, the other variables reflecting temporal myocardial action potential dispersion are influenced by the severity of CHF [3–5, 7, 25, 26]; hence they could fail in SCD-oriented prospective studies. Furthermore, because QT_*p*_-*T*_*e*_ spectral coherence remains uninfluenced by the supraventricular rhythm it might provide useful insight also in patients with atrial fibrillation.

Last, RR-interval power spectral analysis did not find any significant difference between the study groups, except for a lower LH/HF value in the group with Holter ECG-documented sustained VT with respect to the one without. The latter phenomenon is most likely due to the apparently paradoxical reduction in the LF component. Notwithstanding the fact that it might expect LF, a component thought to mirror the sympathetic modulation of the sinus node, to increase, the more the HF syndrome worsens, the more the LF, expressed in the absolute form, decreases, with a partial vagal activity counterbalance [7, 8, 23, 25]. The reduction in LF power in CHF could be linked to reduced responsiveness of the adrenergic beta-receptor, to a loss of oscillatory behavior during chronic sympathetic over activity, and to a sinus node dysfunction [4, 8, 25]. Probably due to the inhomogeneous cohort of patients enrolled in the present study (nearly one half with quite preserved left ventricular ejection fraction), we showed only a trend toward the significance for the LF values reduction whereas the abovementioned mechanisms have been magnified only throughout the LF/HF ratio.

In conclusion, our data suggest that a reduced QT_*p*_-*T*_*e*_ spectral coherence might be a promising noninvasive marker of VT in patients with known structural heart disease and at moderate-to-high risk for SCD. Further prospective studies in larger samples should investigate whether QT_*p*_-*T*_*e*_ spectral coherence can help in stratifying patients with heart failure.

## 5. Limitations

The small study sample, together with the present lack of prospective data, represents an obvious limitation that allows us to suggest, rather than conclude, that QT_*p*_-*T*_*e*_ spectral coherence might have a useful place in improving the screening of those patients yet at moderate-to-high SCD risk. However we believe that our preliminary findings deserve attention, mainly because of the noninvasive, easy, and cheap character of this ECG derived index.

Another consequence of the small study sample is the weight of different diagnosis on the possibility of developing VT. In the group of all ischemic patients (*n*: 106), 23 developed VT (22%) and in the group of patients with primitive cardiomyopathy (*n*: 18), VT arose in 22%. Thus, we have to underline that 85% of patients with VT have an ischemic history, and this point could represent a bias of the study.

Another possible limitation might be represented by the heterogeneous characteristics of patients enrolled in the present study that prevent us from extrapolating our findings to a specific category of cardiac structural disease. In particular, medications (e.g., Amiodarone and Ivabradine) and gender could influence results, although there was no statistical difference between the two groups for these parameters. For the same reason, it could be also hypothesized that mechanisms underlying the poor QT_*p*_-*T*_*e*_ spectral coherence we found in the group with Holter ECG-documented sustained VT might be different. Indeed, an evaluation of the intra-QT spectral coherence implies that we compared the late myocardial repolarization (i.e.,* T*_*e*_) versus the depolarization (*Q-J* interval) plus the early myocardial repolarization phases (*J*-*T*_*p*_). Thus, it is likely that different cardiac diseases (i.e., previous myocardial infarction rather than primitive or postischemic cardiomyopathy) might lead to different ion channels dysfunction. Nevertheless, albeit the abovementioned aspect might be interpreted as a limit of the present research, it might also represent an advantage of this index. Accordingly, in the patients we studied, the reduced QT_*p*_-*T*_*e*_ spectral coherence was associated with VT independently from the other clinical variables, including the left ventricular ejection fraction.

## Figures and Tables

**Table 1:** General characteristics in the two study groups.

Variables	VT group *N*: 27	No-VT *N*: 97	*P* values
Age, yrs	64.3 ± 11.3	59.4 ± 12.4	ns
M/F,	25/2	86/11	ns
BMI, kg/m^2^	25.4 ± 2.4	26.5 ± 3.3	ns
HR, beats/min	64 ± 9.5	63 ± 8.4	ns
SBP, mm Hg	123 ± 25	124 ± 23	ns
DBP, mm Hg	65 ± 10	67 ± 9	ns
EF, %	41 ± 10	38 ± 11	ns
QT_*e* Bazett_, ms	401 ± 57	388 ± 60	ns
QT_*e* Fridericia_, ms	398 ± 55	385 ± 63	ns
QT_*e* Lilly_, ms	399 ± 56	386 ± 62	ns
QT_*e* Framingham_, ms	398 ± 56	386 ± 62	ns
QT_*p* Bazett_, ms	312 ± 51	299 ± 44	ns
QT_*p* Fridericia_, ms	309 ± 49	297 ± 45	ns
QT_*p* Lilly_, ms	310 ± 50	297 ± 44	ns
QT_*p* Framingham_, ms	309 ± 50	298 ± 42	ns
*T*_*e* Bazett_, ms	89 ± 24	92 ± 37	ns
*T*_*e* Fridericia_, ms	89 ± 24	92 ± 37	ns
*T*_*e* Lilly_, ms	89 ± 24	92 ± 37	ns
*T*_*e* Framingham_, ms	93 ± 28	96 ± 62	ns
LVEF, %	41 ± 10	38 ± 11	ns
NYHA class, II/II	10/17	41/56	ns
*β*-Blockers, *n*	20 (74%)	76 (78%)	ns
Furosemide	10 (37%)	51 (53%)	ns
ACEi/sartans	21 (78%)	80 (82%)	ns
Spironolactone	10 (37%)	32 (33%)	ns
Amiodarone	7 (26%)	19 (20%)	ns
Ivabradine	2 (7%)	9 (9%)	ns
Digoxin	3 (11%)	11 (11%)	Ns

Data are expressed as mean ± SD. VT: sustained ventricular tachycardia; M/F: male/female; BMI: body mass index; HR: heart rate; LVEF: left ventricular ejection fraction; NYHA: New York Heart Association.

**Table 2:** Short-term (5-minute) ECG derived data in the two study groups.

Variables	VT group *N*: 27	No-VT *N*: 97	*P *values
QT_*e*_ mean, ms	391 ± 56	381 ± 73	Ns
QT_*e*_ variance, ms^2^	46 [59]	43 [49]	Ns
QT_*p*_ mean, ms	293 ± 52	304 ± 48	Ns
QT_*p*_ variance, ms^2^	47 [54]	35 [52]	Ns
*T*_*e*_ mean, ms	91 ± 39	88 ± 25	Ns
*T*_*e*_ variance, ms	99 [76]	86 [93]	Ns
RR mean, ms	961 ± 142	966 ± 137	ns
RR variance, ms^2^	707 [879]	729 [962]	ns
QT_*e*_ variability index	−0.46 [1.17]	−0.35 [0.80]	ns
QT_*p*_ variability index	−0.21 [1.22]	−0.16 [0.84]	ns
*T*_*e*_ variability index	1.17 0 [1.26]	1.28 [0.79]	ns

Values are expressed as mean ± SD (compared with unpaired *t*-test) or median [interquartile range 75th percentile–25th percentile] (Mann-Whitney *U* test).

**Table 3:** Short-term QT-RR and intra-QT spectral coherence values in the two study groups.

Variables	VT group *N*: 27	No-VT *N*: 97	*P *values
QT_*e*_ → RR, coherence	0.223 ± 0.056	0.218 ± 0.049	ns
QT_*p*_ → RR, coherence	0.236 ± 0.076	0.230 ± 0.061	ns
*T*_*e*_ → RR, coherence	0.229 ± 0.147	0.214 ± 0.037	ns
QT_*e*_ → QT_*p*_, coherence	0.207 ± 0.034	0.233 ± 0.089	ns
QT_*e*_ → *T*_*e*_, coherence	0.504 ± 0.170	0.520 ± 0.195	ns
QT_*p*_ → *T*_*e*_, coherence	0.508 ± 0.150	0.607 ± 0.150	**0.016**

Values are expressed as mean ± SD (unpaired *t*-test).

**Table 4:** Short-term RR spectral data in the two study groups.

Variables	VT group *N*: 27	No-VT *N*: 97	*P *values
TP, ms^2^	733 [1622]	715 [840]	ns
VLF, ms^2^	341 [888]	367 [519]	ns
LF, ms^2^	101 [255]	143 [193]	ns
HF, ms^2^	85 [231]	79 [141]	ns
LF/HF	1.21 [1.00]	1.64 [2.60]	**0.045**

Values are expressed as median [interquartile range 75th percentile–25th percentile]. TP: total power; VLF: very-low-frequency power; LF: low-frequency power; HF: high-frequency power (Mann-Whitney *U* test).
